# Pattern of serum bilirubin changes following double volume exchange blood transfusion in neonates at a tertiary health facility in Nigeria

**DOI:** 10.11604/pamj.2021.39.60.26408

**Published:** 2021-05-21

**Authors:** Olufunmilola Olubisi Abolurin, Adesola Olubunmi Adekoya, Tinuade Adetutu Ogunlesi, Emmanuel Damilare Ajibola, Taiwo Ebunoluwa Adekanye, Eniola Mary Adeniran

**Affiliations:** 1 Department of Paediatrics, Babcock University Teaching Hospital, Ilishan, Ogun State, Nigeria,; 2 Department of Paediatrics, Olabisi Onabanjo University, Sagamu, Ogun State, Nigeria,; 3 Nursing Services Unit, Department of Paediatrics, Babcock University Teaching Hospital, Ilishan, Ogun State, Nigeria

**Keywords:** Acute bilirubin encephalopathy, exchange blood transfusion, neonatal jaundice, serum bilirubin, severe hyperbilirubinaemia

## Abstract

**Introduction:**

exchange blood transfusion (EBT) is a form of massive transfusion useful in rapidly reducing serum bilirubin levels, but serum bilirubin levels frequently rebound within hours of completing the procedure, due to equilibration of extravascular bilirubin as well as on-going hemolysis. The study was carried out to determine the pattern of reduction in serum bilirubin levels following EBT among neonates with severe hyperbilirubinemia, as well as the factors contributing to this pattern, so as to establish evidence-based expectations following EBT.

**Methods:**

a retrospective descriptive study covering a two-year period in a Nigerian tertiary hospital. Details of the EBT procedures, including serial serum bilirubin levels, were obtained from the hospital records of all newborn babies who had double volume EBT done for severe hyperbilirubinaemia during the study period. Data was analyzed using the statistical software SPSS version 21.0.

**Results:**

the mean total serum bilirubin (TSB) before EBT in the 36 babies was 17.9 ± 6.3 mg/dl. The mean percentage decrease in TSB immediately following EBT was 44.3 ± 10.2%. Six hours after EBT, TSB levels had increased from the immediate post-EBT values by an average of 57.5 ± 32.2%. Twenty-four hours after the procedure, TSB values in most (87.1%) cases were still higher than the immediate post-EBT values, but lower than the pre-EBT values. Post-EBT anemia was recorded among 33.3% of the babies.

**Conclusion:**

EBT is effective in rapidly reducing serum bilirubin levels and preventing acute bilirubin encephalopathy in neonates with severe hyperbilirubinemia, despite the rebound increase that occurs in TSB values after the procedure.

## Introduction

Neonatal jaundice is a common medical problem in the first week of life, of which a delay in management could lead to significant morbidity and mortality [1,2]. Phototherapy and exchange blood transfusion (EBT) are the most common treatment modalities used for neonatal jaundice. EBT is indicated in situations where phototherapy proves ineffective in preventing a rise in bilirubin to harmful levels, or when serum bilirubin levels are very high at the point of first contact with the baby [1-4]. The effective use of phototherapy has greatly reduced the need for EBT in both developing and developed countries [3]. However, late presentation is a common occurrence in Nigerian babies, most of whom are delivered outside the hospital, resulting in a high rate of EBT [2,3,5].

EBT rapidly removes bilirubin from the blood, thus reducing the risk of brain damage in babies with severe unconjugated hyperbilirubinaemia [1,2]. Anecdotal reports suggest initial rapid drop of up to 60% in serum bilirubin immediately after EBT, which is frequently followed by an increase as a result of equilibration of extravascular bilirubin as well as on-going hemolysis. Therefore, serum bilirubin levels can rebound 40-60% within hours of completing the procedure [1,4]. In some cases, repeat EBTs may be required for the same indications as the initial exchange [4].

To the best knowledge of the researchers, the pattern of reduction in serum bilirubin following EBT and the factors contributing to the pattern have not been extensively studied, hence this study, so as to establish evidence-based expectations following EBT. This will empower paediatricians to make informed decisions relating to EBT in the care of babies with severe hyperbilirubinaemia, as well as appropriately counsel the frequently anxious parents of babies who require the procedure. Ultimately, the case management and outcome of such babies would be improved upon.

## Methods

This was a retrospective descriptive study that involved neonates admitted into the Special Care Baby Unit (SCBU) of the Babcock University Teaching Hospital (BUTH), Ilishan-Remo, Ogun State, Nigeria, over a two-year period spanning June, 2017 to May, 2019. Babcock University Teaching Hospital is a fast-growing tertiary health facility which provides expert services in several specialties to the host community, neighboring towns and states.

The hospital records of all newborn babies who had double volume EBT for severe hyperbilirubinaemia during the period were retrieved. Relevant information extracted from the records constituted the data for the study. This included the age and sex of the baby, gestational age at delivery, place of birth, birth weight and weight at presentation, duration of jaundice before presentation, cause of jaundice, presence of features of acute bilirubin encephalopathy (ABE) such as abnormal muscle tone, poor feeding/sucking, high-pitched cry and convulsions [6], age at which EBT was done, medications given, as well as investigation results, including packed cell volume before and after EBT, serum bilirubin (SB) before EBT, immediately after EBT as well as at 6, 12 and 24 hours after EBT. Serum bilirubin quantification followed standard procedures, using Randox bilirubin test kits (Randox Laboratories Ltd. United Kingdom). Babies who had EBT for other indications such as severe neonatal anaemia, sepsis, disseminated intravascular coagulation (DIC), were excluded from the study.

The babies were classified as preterm (<34 weeks), near-term (34-36 weeks), term (37-41 weeks) or post-term (≥42 weeks) based on their gestational age at delivery. They were also classified as in-born or out-born based on whether they were born in BUTH or in other places, respectively. Exchange blood transfusion was done for term babies who had total serum bilirubin (TSB) ≥20 mg/dl at any point in time, TSB persistently in the high-risk zone (using a normogram designated for the assessment of risk of severity of jaundice) [7] despite phototherapy, or presence of ABE, while TSB ≥10 mg/dl/ kilogram of body weight was considered an indication for EBT in babies who weighed less than 2.0 kg. Babies who had haematocrit less than 45% in the first week of life were considered anaemic, while those older than seven days were considered anaemic when haematocrit was less than 35%.

Data analysis was done using the Statistical Package for the Social sciences (SPSS) software version 21.0. Appropriate descriptive statistics, including frequencies, means and standard deviations, were used to describe variables, while inferential statistics such as the Student t-test and Pearson´s chi-square (χ^2^) test were used to compare continuous and categorical variables respectively. In the event of missing data, analysis included only participants whose data was available. Probability (p) values less than 0.05 were considered statistically significant. Ethical clearance for the study was obtained from the Babcock University Health Research Ethics Committee (BUHREC/453/19).

## Results

A total of 408 neonates were admitted into the SCBU over the two-year study period, of which 124 (30.4%) had neonatal jaundice (NNJ). Thirty-six (29.0%) of the 124 babies managed for NNJ had double-volume EBT done for severe hyperbilirubinaemia. Twenty-seven (75.0%) of them had only one session of EBT, while 9 (25.0%) had two sessions of EBT. Nineteen (52.8%) of the babies were males, while 17 (47.2%) were females. Eighteen (50.0%) were term babies, 7 (19.4%) were near-term, while 11 (30.6%) were preterm babies; none was post-term. Fifteen (41.7%) of the babies were in-born, while the remaining 21 (58.3%) were out-born. Fourteen (38.9%) of them were admitted within the first 24 hours of life, albeit only two of the 14 (14.3%) were admitted primarily for jaundice. Three (8.3%) were admitted at age >7 days, the oldest being 11 days. Their weights at point of admission ranged from 0.90 to 4.10 kg with a mean (±SD) of 2.36 ± 0.89 kg. The need for EBT was established at the point of admission in sixteen (44.4%) of the babies.

The mean age at which jaundice was first noticed was 2.92 ± 1.93 days. There was a significant difference in the mean age at which jaundice was first noticed between in-born and out-born babies (2.00 ± 1.00 vs 3.57 ± 2.18 days; p = 0.014). It was most frequently noticed on the second day of life (17 babies; 47.2%), while it was noticed on the first day of life in 6 (16.7%) babies. Nine (25.0%) babies, all out-born, already had features of acute bilirubin encephalopathy (ABE) at the time of presentation; these babies were mostly term or near-term, with only one being preterm. None of the other babies developed ABE during the course of management.

The age at which EBT was done ranged from 2 to 13 days, with a mean of 5.00 ± 2.75 days. Top-up whole blood transfusion was given to all the babies after completion of double-volume EBT; the volume of top-up transfusion was 10 ml/kg in 23 (63.9%) babies, 20 ml/kg in 8 (22.2%) and <10 ml/kg (due to insufficient blood) in 5 (13.9%) babies. All the babies received phototherapy as an adjunctive treatment both before and after EBT. Thirty-four (34) babies had serum SB done immediately after completion of EBT, SB was repeated six hours after EBT in 22 babies, 12 hours after EBT in 17 babies and 24 hours after EBT in 33 babies.

The documented causes of jaundice are highlighted in Table 1. Sepsis was the most common cause, followed by jaundice of prematurity. Pre-EBT TSB ranged from 7.50 to 33.70 mg/dl with a mean of 17.9 ± 6.3 mg/dl. The mean was 13.59 ± 3.78 mg/dl among those who were preterm and 19.81 ± 6.27 mg/dl among the term and near-term babies. The conjugated fraction of the pre-EBT bilirubin ranged from 0.80 - 4.64 with a mean of 2.18 ± 0.92 mg/dl. Most (9/13; 69.2%) of the babies who had a conjugated fraction of bilirubin >2.0 mg/dl had sepsis. Post-EBT reduction in TSB was observed in all the 34 babies who had immediate post-EBT SB done (mean of 17.8 ± 6.4 mg/dl pre-EBT vs 9.9 ± 4.0 mg/dl post-EBT). The percentage reduction in TSB ranged from 26.7% - 64.5% with a mean reduction of 44.3 ± 10.2%. The percentage reduction in TSB was not significantly associated with higher pre-EBT values as shown in Table 2. Likewise, the percentage reduction was not significantly different between preterm, near-term and term babies (46.3 ± 11.9%, 42.2 ± 7.8% and 43.9 ± 10.2%, respectively; F= 0.308, p = 0.737). There was also a reduction in the conjugated fraction of bilirubin (CB) in 31 of the 34 babies who had immediate post-EBT SB done with a mean percentage reduction in CB of 38.4 ± 15.4%.

**Table 1:** identified aetiologic factors of neonatal jaundice among babies who had exchange blood transfusion

Aetiologic factor	Number of babies (%)
Sepsis	12 (33.3)
Jaundice of prematurity*	10 (27.8)
ABO blood group incompatibility	6 (16.7)
Glucose-6-phosphate dehydrogenase (G6PD) deficiency	5 (13.9)
ABO incompatibility + G6PD deficiency	2 (5.6)
ABO + rhesus incompatibility	1 (2.8)
Total	36 (100.0)

* Four of the babies with jaundice of prematurity also had confirmed sepsis

**Table 2:** relationship between pre-EBT TSB values and the percentage reduction in TSB following EBT

Pre-EBT TSB range (mg/dl) [n = number]	Mean pre-EBT TSB (mg/dl)	Mean Immediate post-EBT TSB (mg/dl)	Mean reduction in TSB (mg/dl)	Mean percentage reduction in TSB (%)
≤ 10.0 (n = 3)	8.7 ± 1.1	4.8 ± 1.5	3.9 ± 0.6	46.0 ± 11.6
>10.0 but ≤15.0 (n = 10)	13.1 ± 1.1	7.8 ± 1.4	5.3 ± 1.3	40.3 ± 9.3
>15.0 but ≤20.0 (n = 11)	17.0 ± 1.6	8.9 ± 1.9	8.2 ± 1.8	47.9 ± 10.7
>20.0 (n = 12)	25.1 ± 4.6	14.1 ± 3.8	11.1 ± 3.1	44.3 ± 10.3
				F = 0.961, p = 0.424

EBT: exchange blood transfusion; TSB: total serum bilirubin

By six hours post-EBT, TSB levels had increased from the immediate post-EBT values in all the 20 babies who had both immediate post-EBT SB and 6hr-post-EBT SB recorded (mean of 9.2 ± 2.8 mg/dl immediately after EBT vs 14.1 ± 3.7 mg/dl after 6 hours). The mean percentage rise in TSB within six hours post-EBT was 57.5 ± 32.2%. Twelve hours post-EBT levels were higher than immediate post EBT values in most (15/17; 88.2%) babies, with only 2 (11.8%) babies having lower TSB levels compared with immediate post-EBT values. By 24 hours after EBT, TSB values (mean of 12.7 ± 3.2 mg/dl) were still higher than immediate post-EBT values in most (87.1%) babies, but the 24hr-post-EBT values were mostly (87.9%) lower than the pre-EBT values. Complications of EBT were noted in 12 (33.3%) of the 36 babies who had the procedure done. The 12 babies had post-EBT anaemia, although 6 (50.0%) of them were already anaemic prior to EBT. In addition, 2 of the 12 babies developed omphalitis following EBT. On the other hand, pre-EBT anaemia was corrected by the EBT in 10 (62.5%) of all the 16 babies that had anaemia prior to EBT. Although the prevalence of post-EBT anaemia appeared to be higher in those who received lower volumes of top-up transfusion, the difference was not statistically significant as shown in Table 3. Hypoglycaemia was not reported in any of the babies following EBT.

**Table 3:** relationship between volume of top-up transfusion and the occurrence of post-EBT anaemia

Volume of top-up transfusion	Post-EBT anaemia	
	**Yes n (%)**	**No n (%)**
<10 ml/kg	3 (60.0)	2 (40.0)
10 ml/kg	7 (30.4)	16 (69.6)
20 ml/kg	2 (25.0)	6 (75.0)
Total	12 (33.3)	24 (67.7)
Statistical significance	χ2 = 1.834, p = 0.400	

EBT: exchange blood transfusion

There was a mean percentage reduction of 51.7 ± 5.4% in TSB, with a range of 39.9 - 58.1% following the second EBT in the 9 babies who had two EBTs done. The pre-EBT and post-EBT mean TSB values for the second EBT were 17.5 ± 3.6 mg/dl and 8.4 ± 1.8 mg/dl, respectively. No death was recorded among the babies studied; 35 (97.2%) of them were discharged when stable, while 1 (2.8%) was discharged against medical advice.

## Discussion

The purpose of performing an EBT in a severely jaundiced baby is to urgently reduce the blood level of unconjugated bilirubin so as to prevent or minimize brain damage [1,2]. In the present study, EBT achieved this purpose with more than 40 percent immediate reduction in TSB. This supports previous reports of the effectiveness of EBT in rapidly reducing bilirubin levels [5,8-10]. However, bilirubin levels were found to have rebounded by more than half the immediate post-EBT values within six hours. This also falls within expectations based on previous reports [1,4]. Ambalavanan and Carlos reported that bilirubin levels may rebound 40-50% within six hours following EBT [1], while Gregory *et al*. predicted as high as 60% rebound values [4]. The differential efficiency of phototherapy devices may contribute to these differences. Nevertheless, following the rebound increase, TSB levels reduced gradually, resulting in lower values after 24 hours, compared with the six-hour levels.

Although the TSB levels at 24 hours were still much higher than the immediate post-EBT levels, they were less than the pre-EBT values. The resulting lower post-EBT bilirubin levels reduced the risk of ABE in the concerned babies, coupled with the fact that each additional day of life lowers the risk/severity of a particular TSB level. This is exemplified by the fact that a TSB level of 12 mg/dl is considered high-risk in babies aged ≤40 hours, intermediate-risk in babies aged 40 - 90 hours, but low-risk in babies aged >90 hours [7]. Therefore, it can be inferred that even with the rebounding pattern of TSB levels after EBT, the procedure offers significant protection against acute bilirubin encephalopathy (ABE). Furthermore, none of the babies who did not have features of ABE *ab initio* developed such features during management, confirming that EBT was effective in preventing ABE.

All nine babies who had ABE were out-born and the features were already evident at the point of admission. This finding supports the observation made by Owa *et al*. that the persistently high rate of EBTs in Nigeria is mainly due to late presentation at the hospital [3]. Sadly, two of the babies who had ABE in the present study were part of a set of triplets; the first of the triplets presented with inability to suck and was severely jaundiced, but died while preparations were being made for EBT, which prompted a request for the other two to be brought to the hospital for review. Surprisingly, these other two, whom the family did not consider ill enough to require hospital care, turned out to also be severely jaundiced with signs of ABE and they had EBT done urgently. Insufficient counseling of mothers on detection of neonatal jaundice at home as well as inadequate follow-up of babies in the first week of life, coupled with wrong and ineffective treatments being given to many jaundiced babies, are likely contributors to this problem [11]. The apparent lower prevalence of ABE among preterm babies is likely because most preterm babies would require special care for other problems such as hypothermia and respiratory distress syndrome very early in life before jaundice becomes severe in them [12].

The most common causes of jaundice in the present study were sepsis and prematurity. Generally, the aetiological pattern observed was similar to those reported in previous studies among babies who had EBT for hyperbilirubinaemia [3,5,8,13]. However, the prevalence of ABO blood group incompatibility (19.5%) was lower than that observed by Pius and Mava [5] (56.7%), Owa and Ogunlesi [3] (30.0%), Onyearugha *et al*. [13] (23.8%) and Ibekwe *et al*. [8] (20.0%), while the prevalence of sepsis was higher than in most of those studies. Only one baby had rhesus blood group incompatibility, which is not surprising considering the low prevalence of the problem among Africans [14]. It is also not surprising that sepsis was confirmed in close to half of the preterm babies, since sepsis is known to be a common problem in such babies [12].

Anaemia was a common complication of EBT in the present study. Although EBT corrected anaemia in a good number of patients, some previously anaemic babies remained anaemic even after EBT. Giving top-up blood transfusion helps to reduce the risk of post-EBT anaemia in newborns. However, the difference in prevalence of post-EBT anaemia with varying volumes of top-up was not statistically significant in the present study, though the pattern observed appeared to favour the use of 20 ml/kg. Further studies to investigate the correction of pre-existing anaemia by EBT, using other study models, will be worthwhile. The absence of EBT-related mortality in the present study is reassuring, compared with previous studies which reported mortality rates ranging from 1.9 to 17.5 percent in babies who had EBT [3,5,8,13,15].

## Conclusion

Exchange blood transfusion is effective in rapidly reducing serum bilirubin levels and preventing ABE in neonates with severe hyperbilirubinaemia, despite the rebound increase that occurs in SB values after the procedure. Additionally, there should be concerted efforts at educating expectant mothers on early identification of neonatal jaundice and prompt presentation in a hospital for evaluation.

Limitations of the study: 1) being a retrospective study, incomplete results were a limitation; 2) the full range of post-EBT complications could not be assessed.

### What is known about this topic

- Exchange blood transfusion (EBT) reduces the risk of brain damage in babies with severe jaundice;
- Serum bilirubin levels usually rebound within few hours after EBT.

### What this study adds

- Despite more than 50% rebound increase in serum bilirubin levels after EBT, the levels mostly remain lower than pre-EBT levels and acute bilirubin encephalopathy is effectively prevented.
